# Lateral gene transfer of streptococcal ICE element RD2 (region of difference 2) encoding secreted proteins

**DOI:** 10.1186/1471-2180-11-65

**Published:** 2011-04-01

**Authors:** Izabela Sitkiewicz, Nicole M Green, Nina Guo, Laurent Mereghetti, James M Musser

**Affiliations:** 1 Center for Molecular and Translational Human Infectious Diseases Research, The Methodist Hospital Research Institute, and Department of Pathology, The Methodist Hospital, Houston, Texas, 77030, USA; 2 Department of Epidemiology and Clinical Microbiology, National Medicines Institute. Warszawa, Poland; 3 Université François-Rabelais, Faculté de Médecine, EA 3854 "Bactéries et Risque Materno-Foetal", Tours, France

## Abstract

**Background:**

The genome of serotype M28 group A *Streptococcus *(GAS) strain MGAS6180 contains a novel genetic element named Region of Difference 2 (RD2) that encodes seven putative secreted extracellular proteins. RD2 is present in all serotype M28 strains and strains of several other GAS serotypes associated with female urogenital infections. We show here that the GAS RD2 element is present in strain MGAS6180 both as an integrative chromosomal form and a circular extrachromosomal element. RD2-like regions were identified in publicly available genome sequences of strains representing three of the five major group B streptococcal serotypes causing human disease. Ten RD2-encoded proteins have significant similarity to proteins involved in conjugative transfer of *Streptococcus thermophilus *integrative chromosomal elements (ICEs).

**Results:**

We transferred RD2 from GAS strain MGAS6180 (serotype M28) to serotype M1 and M4 GAS strains by filter mating. The copy number of the RD2 element was rapidly and significantly increased following treatment of strain MGAS6180 with mitomycin C, a DNA damaging agent. Using a PCR-based method, we also identified RD2-like regions in multiple group C and G strains of *Streptococcus dysgalactiae *subsp.*equisimilis *cultured from invasive human infections.

**Conclusions:**

Taken together, the data indicate that the RD2 element has disseminated by lateral gene transfer to genetically diverse strains of human-pathogenic streptococci.

## Background

We sequenced the genome of a strain (MGAS6180) of serotype M28 group A *Streptococcus *[1], a human-specific pathogen that is non-randomly associated with neonatal female urogenital infections [2]. The genome of strain MGAS6180 has a novel 37-kb element designated RD2 (Region of Difference 2) [1]. RD2 is one of seven elements integrated into the chromosome of this strain (4 phages, 3 ICE and ICE related elements) [1,3]. Subsequently we demonstrated that all serotype M28 strains studied contained RD2 integrated at the same chromosomal site [1,3]. RD2 encodes seven secreted extracellular proteins that are expressed in human infections. One of these proteins (M28_Spy1336) is also known as the R28 protein, and has been previously studied in GAS and group B *Streptococcus *(GBS) [4-7]. The R28 protein has been implicated in virulence based on its ability to mediate binding of GBS to human vaginal epithelial cells [6]. Protein M28_Spy1325 was recently studied extensively and shown to be a member of the antigen I/II family of proteins originally described in oral streptococcal species. M28_Spy1325 binds to salivary agglutinin, a 340-kDa protein abundantly found in human saliva. Zhang et al. [8] recently demonstrated that immunization of mice with recombinant purified M28_Spy1325 confers protection against invasive infection. Thus, two proteins encoded by RD2 likely contribute to host-pathogen interactions.

Several lines of evidence suggest that RD2 in GAS was acquired by horizontal gene transfer (HGT). First, the RD2 element is integrated into a tRNA-threonine gene and flanked by 16bp imperfect direct repeats ATTC(C/T)CGGTGGTGGCA [1,3]. The chromosomal location of RD2 is identical in the majority of RD2-positive strains suggesting a conserved mode of integration [1]. Second, the G+C content of RD2 (35%) is significantly lower than the average GAS genome (38%) and contains different di-nucleotide content and codon usage [1,3]. An RD2-like element also has been identified in the genome of a serotype M2 GAS strain [3]. The RD2 element in this strain is virtually identical at the nucleotide level to RD2 present in M28 strains. However, the genome sequence of strain MGAS6180 (M28) and MGAS10270 (M2) are otherwise quite divergent from one another. Based on single nucleotide polymorphism (SNPs), the average SNP difference between genomes is about 137 per 1 kb (total of 14096 SNPs), while only 8 nucleotide differences are found within 37 kb RD2 region [3,9]. The differences in SNP frequency within chromosome and RD2 region strongly suggests that the RD2 element in these strains has had a very different evolutionary history compared to the core chromosome, and was acquired via horizontal transfer [3].

The primary goal of the experiments described herein was to test the hypothesis that the RD2 element was laterally transferable *in vitro *under laboratory conditions, and we found that this was the case. Moreover, we identified an RD2-like element in multiple strains of Lancefield group C and G streptococci, indicating that this genetic element is more phylogenetically widespread than previously appreciated.

## Methods

### Bacterial strains and growth

Streptococcal strains of serotypes A, C, and G (Additional File 1, Table S1) were cultured routinely at 37°C in an atmosphere of 5% CO_2 _on Trypticase soy agar II plates containing 5% sheep blood (Becton Dickinson, Franklin Lakes, NJ) or in liquid Todd Hewitt medium supplemented with 0.5% yeast extract (THY medium). Antibiotics were used at the following concentrations: spectinomycin, 150 μg/ml; erythromycin, 1 μg/ml; and kżanamycin, 400 μg/ml.

### Isolation of total DNA from streptococci

DNA was isolated from cultures grown overnight in THY medium using a modified phenol-chloroform procedure [10]. Briefly, 5 to 35 ml of overnight THY cultures were pelleted by centrifugation and suspended in TE, pH 7.5. Bacteria were treated with mutanolysin (500 U/ml) and lysozyme (2 mg/ml) (Sigma Aldrich, St. Louis, MO) with occasional mixing for 1 h at 37°C, followed by 2% wt/vol SDS (Gibco, Carlsbad, CA), and proteinase K (0.2 mg/ml) (Sigma Aldrich, St. Louis, MO) for 1 h at 37°C. DNA was extracted with phenol:chloroform:isoamyl alcohol, precipitated with two volumes of ice-cold ethanol, washed with 70% ice-cold ethanol, and suspended in TE, pH 8.0 buffer containing 0.2 mg/ml RNase A (Invitrogen, Carlsbad, CA).

### DNA amplification by PCR

Primers used in PCR reactions are listed in Table 1 and Additional File 2 (Table S2). PCR was used to investigate the presence and organization of the RD2 element in streptococcal strains. The PCR primers #1-#4 detect a chromosomal and extrachromosomal circular form, and tile across the entire RD2. Confirmation of RD2 presence by tailing and detection of genes encoding extra-chromosomal proteins was performed as described previously [1,2]

**Table 1:** PCR primers used in this study

**A**. Primers used for detection of multiple RD2 genes, Q-PCR and tiling.		
**Primer name**	**Primer sequence**	**Source**

***emm *sequencing**		

CDC emm1	TATT(C/G)GCTTAGAAAATTAA	[19]

CDC emm2	GCAAGTTCTTCAGCTTGTTT	[19]

**Detection of circular form**		

#1	GAAAACAAAAGTTTCTTCATGCGTTTGGCG	this work

#2	CAATTAATAGAAACATATGGTCATTTG	this work

#3	GGAATTAGCCCACTAGAATATAAGC	this work

#4	TAGCAAGTAAACCCTAGATTGTCTATGTTC	this work

**Detection of genes encoding extracellular RD2 proteins**		

M28_Spy1306F	ACTAAGCCAAGCGAGGACAA	[1]

M28_Spy1306R	CCAAAACCGTGTAGCCTGTA	[1]

M28_Spy 1307F	TCATCGTCAAAAGCCATCTC	[1]

M28_Spy 1307R	TTGCTCTGATAAACCTCAAG	[1]

M28_Spy1308F	TACGACAGAAGCAGGTGGAG	[1]

M28_Spy1308R	ACCGAGTTTCGCAGGATTG	[1]

M28_Spy1325F	TGAATGATGCGGGGACTTAT	[1]

M28_Spy1325R	TGTAAAAGGCTGCTGGGTCT	[1]

M28_Spy1326F	ACACCGACTGAGATTGCTGA	[1]

M28_Spy1326R	TTGGCTTGTGAGGTTTGAGA	[1]

M28_Spy1332F	ATGCCAAAAACCAAAGGAAG	[1]

M28_Spy1332R	TCATACTTTTCAGGTACACAAGCA	[1]

M28_Spy1336F	GATACTTCACAGACGAAACAACG	[1]

M28_Spy1336R	ATCACGACTCCCATCACTCC	[1]

**Quantitative PCR (Taqman)**		

proS_F	TGAGTTTATTATGAAAGAGGCTATAGTTTC	[15]

proS_R	AATAGCTTCGTAAGCTTGACGATAAT	[15]

proS_P	TCGTAGGTCACATCTAATCTTCATAGTTG	[15]

M28_Spy1306 F	CGTTGTTCCTGCTACTGGATCTGCTAC	this work

M28_Spy1306 P	ACGATTGCAAGTATTGCTTTG	this work

M28_Spy1306 R	CAATCGGTGTCGTTGGTTG	this work

M28_Spy1325 F	ACCGTCGCAAGGACCTTGTCTTTCTG	[8]

M28_Spy1325 P	CAGCATACGCATGACCTC	[8]

M28_Spy1325 R	AGTGATAACACTACCATCTGATAAAG	[8]

M28_Spy1336 F	ACAGAAGCTGCACCAAACTTGAACTTCTTAATTGA	this work

M28_Spy1336 P	GTAGATGCAGCAACTATTGAC	this work

M28_Spy1336 R	ATGATACTTCACAGACGAAACAAC	this work

M28_Spy0784_RD0 F	AGCAGAGTATGAAGGCGGTTTT	this work

M28_Spy0784_RD0 P	ATATTCTATCTGAAACGGCTCG	this work

M28_Spy0784_RD0 R	AACATCTCTGCGAGTCGTTCTATACTT	this work

M28_Spy0980_6180.1 F	TCGTTAGGACTGGCGGTAGAG	this work

M28_Spy0980_6180.1 P	TGCAACTGCTGTCTTAA	this work

M28_Spy0980_6180.1 R	AACAGTCTTTGCCACCACCAT	this work

M28_Spy1087_RD1 F	TGTTTTTTGAATCTCTGACTTCTTTCC	this work

M28_Spy1087_RD1 P	AGAATTGCAGCTACTTGTATT	this work

M28_Spy1087_RD1 R	TGCAGACGAAAATAGCTGTAACTACTC	this work

M28_Spy1231_6180.2 F	GCAGTTGCTTGTTGCGTTGA	this work

M28_Spy1231_6180.2 P	TGCAACCCACTGATTT	this work

M28_Spy1231_6180.2 R	GCGCGTAGAGCTGGAGTCA	this work

M28_Spy1805_6180.3 F	AAAGGGCTATGGACGAACGA	this work

M28_Spy1805_6180.3 P	CAGACCAGCCTTTG	this work

M28_Spy1805_6180.3 R	GGTAAACCGATATTTTTCATCAATGA	this work

**B**. Primer combinations used for tiling across RD2 element, after [1].		

**Tiling fragment**	**Amplified region**	**Primer sequence**

1	M28_Spy1299-1304	GGTTTCGACAAGGTCAGAGC
		
		TGTGAGTGTTCCTGTACCAGATG

2	M28_Spy1304-1306	ACGGCTACCTTTCCCCCTA
		
		ACTAAGCCAAGCGAGGACAA

3	M28_Spy1306-1307	CCAAAACCGTGTAGCCTGTA
		
		TCATCGTCAAAAGCCATCTC

4	M28_Spy1307-1308	TTGCTCTGATAAACCTCAAG
		
		TACGACAGAAGCAGGTGGAG

5	M28_Spy1308-1310	ACCGAGTTTCGCAGGATTG
		
		GCTTGGAGGTGTTTCCTTTC

6	M28_Spy1310-1314	CCTTGTTCTGCTTGATGTCC
		
		ATCAAGCAAGCAACAAAACG

7	M28_Spy1314-1322	TTTCCACCCATCAGTTCAGG
		
		GACTGGTGGCGGTAAGACTG

8	M28_Spy1322-1325	TTTCATCCCCAAAAAGCATC
		
		TGAATGATGCGGGGACTTAT

9	M28_Spy1325-1326	TGTAAAAGGCTGCTGGGTCT
		
		ACACCGACTGAGATTGCTGA

10	M28_Spy1326-1331	TTGGCTTGTGAGGTTTGAGA
		
		TCATACTTTTCAGGTACACAAGCA

11	M28_Spy1331-1336	ATGCCAAAAACCAAAGGAAG
		
		GATACTTCACAGACGAAACAACG

12	M28_Spy1336-1338	ATCACGACTCCCATCACTCC
		CAAAGTTCCTGCCCCAAC

### Construction of isogenic mutant strain MGAS6180Δ1325-1326spc^R^

Allelic replacement was used to construct an isogenic mutant strain in which two contiguous genes (M28_Spy1325 and M28_Spy1326) encoded by RD2 were deleted and replaced by spectinomycin resistance cassette [11]. Upstream and downstream regions flanking the two-gene segment were cloned in pTOPO plasmid (Invitrogen) with spectinomycin resistance cassette between them. The gel purified PCR product encompassing both flanks with the spectinomycin cassette was electroporated into cells of strain MGAS6180 made competent as described before [12]. The resulting isogenic strain was confirmed to be the correct construct by PCR analysis, DNA sequencing, and Southern hybridization. Successful inactivation of the *Spy1325 *and *Spy1326 *genes also was confirmed by quantitative real-time PCR and Western immunoblot analysis. Detailed strain construction is presented as Additional File 3 and the confirmation of the proper construction as Additional File 4 (Figure S1).

### Filter mating

Filter mating procedure was performed according to modified method described previously [13]. The MGAS6180Δ1325-1326spc^R ^strain was used as a donor of the RD2 element in filter mating experiments. Strains MGAS2221ΔcovRS (M1, kanamycin resistance, RD2^neg^; P. Sumby unpublished), and MGAS10750 (M4 serotype, natural erythromycin resistance, RD2^neg^; [9]) were used as recipient strains. Overnight donor and recipient cultures (750 μl of each) were mixed and collected on the surface of a 0,45 μm pore size sterile nitrocellulose filter (Millipore). The filter was transferred to the surface of TSA plate without antibiotics and incubated for 3 h, 6 h, or 16 h. After the incubation, the filter was washed with sterile PBS and the bacteria collected in the wash fluid were plated on THY agar plates with appropriate antibiotics. Randomly selected colonies resistant to both antibiotics were screened by PCR for the size of *emm *gene amplicon that is characteristic for M28 or M4 type and presence of RD2 region genes.

### Induction of genetic elements with mitomycin C and hydrogen peroxide

Genetic elements were induced by treating bacterial cultures with mitomycin C as described previously [14]. Briefly, 750 ml of pre-warmed THY medium was inoculated with an overnight culture of MGAS 6180 (1:50 dilution) and grown until the OD reached 0.15 (early log phase). The culture was divided into three aliquots, and one aliquot was treated with mitomycin C (Sigma, final concentration 0.2 μg/ml), second with hydrogen peroxide (final concentration 0,5 mM) and one aliquot was left untreated as a control sample. The concentration of mitomycin C and hydrogen peroxide used for induction of mobile genetic elements was tested for the ability to induce mobile elements and inhibit growth (Additional File 3). The concentrations used in the experiment were sufficient to induce mobile elements in MGAS6180 and were above the minimal inhibitory concentration (Additional File 5, Figure S2). Samples (35 ml) collected at 1 h, 2 h, and 3 h intervals and after overnight incubation and were used for total DNA isolation as described above.

### Quantitative analysis of changes in gene copy number

Total DNA isolated from control GAS or cells treated with mitomycin C or hydrogen peroxide was used as a template in quantitative PCR (Taqman) reactions. Diluted DNA was amplified in multiplex reactions. The primers used amplified the chromosomal gene *proS *(internal calibrator [15]) and the target test gene of interest. Gene copy number was presented as the difference in amplified copies between control gene *proS *and the gene of interest (2^ΔCt^) at each experimental condition. The increase in copy number between start (T0, sample collected immediately prior splitting the cultures and the induction) and time point of interest (T^exp^; e.g. 1 h after the induction) was calculated according to the equation 2^ΔCt TExp^/2^ΔCt T0^.

## Results

### Comparative analysis of RD2 gene content and organization in GAS and GBS

**Figure 1:**
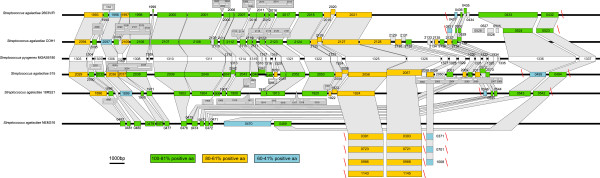
**Comparison of the organization of RD2 ORFs in diverse GAS and GBS strains**. Slanted red lines indicate discontinuity between fragments homologous to RD2 element present in MGAS6180. RD2 open reading frames are marked as white rectangles, open reading frames of GBS are color coded according to their homology to RD2 on the protein level. Numbers within each rectangle represent ORF designation number in particular GBS strain

Sequences homologous to RD2 were initially reported to be present in strains of serotype III and V Group B *Streptococcus *(GBS) [1]. By analyzing the available GBS genomic sequences a number of sequences homologous to RD2 can be identified (Figure 1) [16,17]. The RD2 region in GAS is integrated into gene encoding tRNA for threonine, while elements found in GBS genomes carrying RD2 gene homologs are integrated into gene encoding tRNA for threonine as in GAS, but also tRNA for lysine [17]. Interestingly, the organization of RD2 like element in GBS is strain dependent. In case of strains 515 and COH1, almost the entire element is organized as in MGAS6180 with the exception of *rib *and its regulator (homologs of M28_Spy1336 and M28_Spy1337), whereas in other strains only fragments homologous to RD2 are detected. The regions in *S. agalactiae *genomes homologous to genes M28_Spy1303- M28_Spy1325 are located in majority of analyzed GBS strains within single chromosomal location, while genes M28_Spy1326-M28_Spy1337 are located in other chromosomal location or locations (Figure 1 and Additional File 6, Table S3).

### RD2 encodes a putative conjugation module

**Figure 2:**
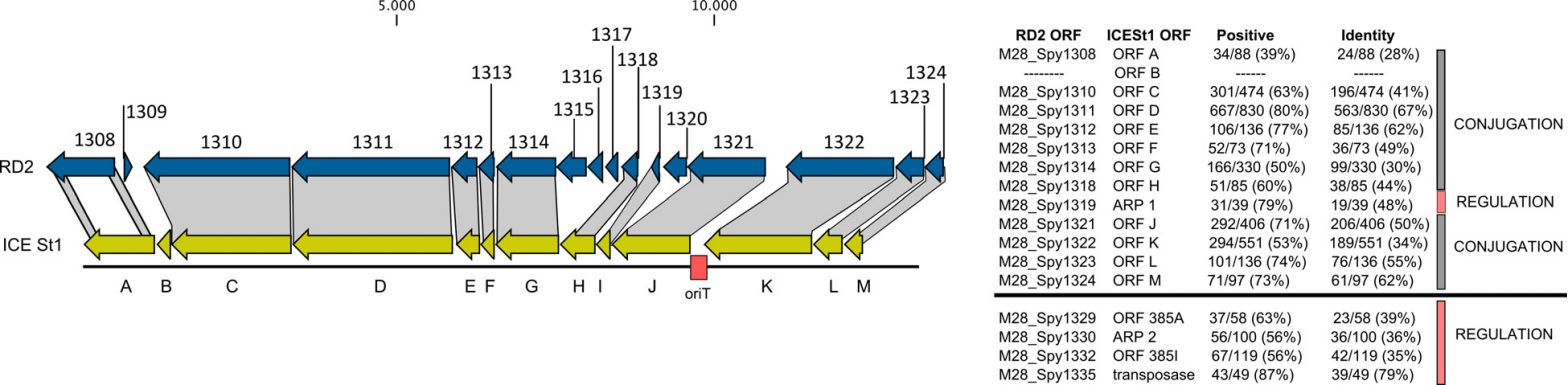
**RD2 encodes homologues of conjugative transfer genes present in the ICE*St1 *and ICE*St3 *elements of *S. thermophilus***.

**Figure 3:**
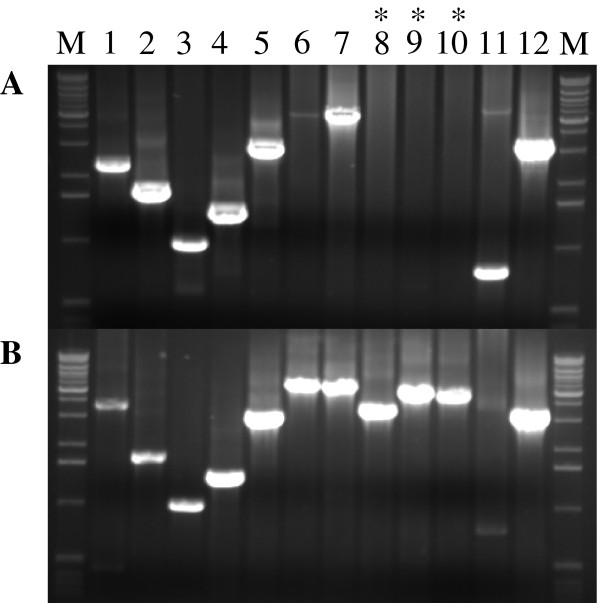
**Detection of RD2 transfer from donor strain MGAS6180 (*emm28*) to recipient strain MGAS10750 (*emm4*)**. Amplicons 1-12 generated by PCR tiling across the RD2 element. **A**. transconjugant; * denote amplicons encompassing deleted M28_Spy1325-1326 region that is replaced by spectinomycin resistance cassette; **B**. control with chromosomal DNA isolated from strain MGAS6180. M - 1 kb ladder (Invitrogen)

Based on DNA sequence analysis, RD2 does not appear to encode genes involved in replication as a circular plasmid. GAS is not considered to be naturally transformable under standard laboratory growth conditions, suggesting that other mechanisms must be used to transfer RD2-related genes between cells. DNA sequence analysis identified a putative transfer module encoded by RD2 with similarity to the ICE*St1 *and ICE*St3 *conjugation modules present in *Streptococcus thermophilus *(Figure 2) [18]. Thus, we hypothesized that RD2 uses a conjugation-like mechanism to transfer from donor to recipient strains. To test this hypothesis, we performed filter mating using donor strain MGAS6180Δ1325-1326spc^R^, which contains a spectinomycin resistance cassette integrated into the chromosomal copy of RD2. The recipient strain used was strain MGAS10750, a type *emm4 *organism that is naturally resistant to erythromycin. After filter mating of strain MGAS6180Δ1325-1326spc^R ^and strain MGAS10750 for 3 h, 6 h, and 16 h, we obtained 1, 3, and 202 colonies, respectively (transfer frequency ~10^-6 ^of transconjugants per donor cell), which were resistant to both antibiotics (spectinomycin 150 μg/ml and erythromycin 1 μg/ml). Eight putative transconjugant colonies were tested for the presence of the RD2 element and characterized for the *emm *gene sequence. In group A *Streptococcus*, *emm *gene is highly polymorphic in sequence and encodes for major surface protein M that is responsible for GAS serotype. Amplification of hyper-variable region of *emm *gene with primers CDCemm1 and CDCemm2 yields products that differ in size depending on the M serotype [19]. RD2 positive transconjugants were first screened based on the *emm *amplicon size (data not shown), and the amplified product was sequenced to confirm that transconjugants belong to M4 serotype, the same as the recipient. Successful transfer of the RD2 element from the *emm28 *strain to the *emm4 *strain was confirmed by PCR tiling across the entire RD2 element (Figure 3). Moreover, based on amplification of the junction region between RD2 and the chromosome, the RD2 element in the transconjugate was integrated at the same locus as in the donor *emm28 *strain (Figure 3). Based on analysis of 43 colonies resistant to both spectinomycin and kanamycin, similar results were obtained using strain serotype M1 strain as the recipient strain (MGAS2221ΔcovRS, resistant to kanamycin).

### RD2 is present in multiple, likely extrachromosomal, copies in GAS

**Figure 4:**
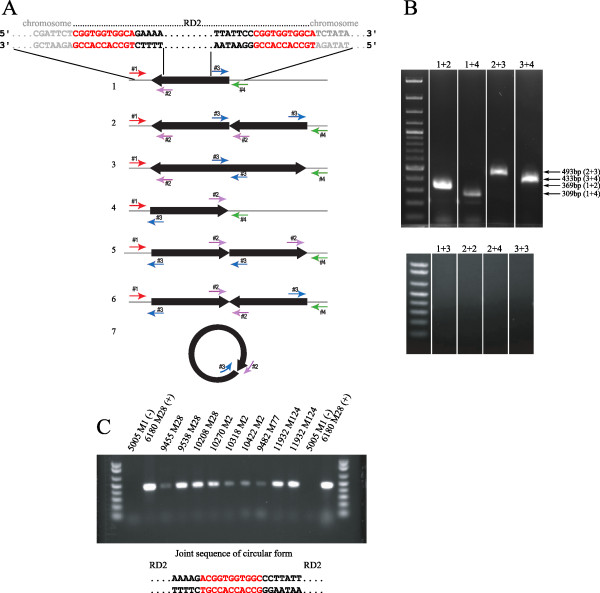
**PCR screen detects multiple or circular copy of RD2**. **A**. Primer combinations used for detection of seven potential arrangements of RD2. Thick black arrows represent RD2 element; thin gray line represents the chromosome. Red, purple, blue, and green arrows represent locations of primer #1, #2, #3, and #4, respectively. Potential arrangement variants are: arrangement as determined by genome sequencing [1], at least two head-to-tail copies of RD2, tail-to-tail, single copy arranged in reverse orientation than the integrative copy determined by genome sequencing, head-to-tail arrangement of copies in reverse orientation than the integrative copy determined by genome sequencing, head-to-head, and circular form. **B**. PCR screen detects product amplified with primer pairs #1+#2, #3+#4, and #2+#3, corresponding to arrangement variant (head-to-tail) or (circular form), and #1+#4 detecting chromosomal integration site lacking RD2. **C**. Primers #2+#3 detect arrangement variant 2 or 7 in multiple RD2 positive strains [1]. Serotype M1 strain MGAS5005 (lacks RD2) was used as a negative control of amplification.

Many gene transfer processes, including conjugation, require circular form of the transferred molecule or that more than one copy of the element exists during at least one point in the transfer cycle [20-22]. Therefore, we tested the hypothesis that multiple copies of the RD2 are present in the bacterial cell. PCR primers were used that allow detection of a circular form of RD2, and permit assessment of the orientation of chromosomal integration of multiple copies of RD2 (Figure 4A). Primers #1 and #4 recognize chromosomal sequences, whereas primers #2 and #3 recognize RD2 element sequences. Depending on the direction and/or arrangement of multiple copies of RD2 (i.e., head-to-head, tail-to-tail, head-to-tail), the different primer combinations would yield distinct amplicons. Based on the genome sequence of strain MGAS6180 [1] primer pairs #1-#2 and #3-#4 would amplify the junction region between the chromosome and RD2 on the left and right flank, respectively (positive control reactions). Using total DNA isolated from an overnight culture of MGAS6180 as template, PCR analysis yielded products amplified with primers #1-#2 and #3-#4, as expected. However, we also observed that primers #2 and #3 amplified a product, a result suggesting the presence of either multiple integrated copies of RD2 or a circular form of RD2 (Figure 4B). Next, we analyzed nine other GAS strains of multiple M protein serotypes using primers #2-#3 to determine if this was a general phenomenon. Regardless of *emm *type, all RD2-positive strains yielded an amplicon with the primer #2-#3 combination whereas RD2-negative organism did not (Figure 4C). Further, DNA sequence analysis revealed that all PCR amplicons generated with primers #2-#3 contained the sequence CGGTGGTGGCA, corresponding to a junction between the left and right flanking regions of RD2 (Figure 4).

**Figure 5:**
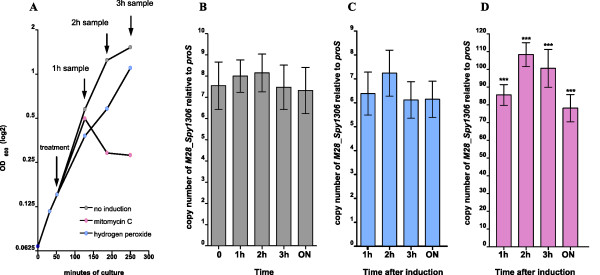
**Mitomycin C treatment results in amplification of RD2**. **A **Rapid decrease in O.D. of a liquid culture of strain MGAS6180 after mitomycin C addition. The decreased O.D. is likely due to prophage induction followed by lytic cycle phage release. Smaller drop in OD is observed after treatment with hydrogen peroxide. **B**. The RD2 element is present in 6-9 copies per chromosome in the absence of inducer. **C**. The RD2 element is not induced by oxidative stress. Bars in each group represent the RD2 copy number after 1 h, 2 h, 3 h, and 16 h after treatment with hydrogen peroxide. **D**. RD2 is induced by DNA damage. Bars in each group represent the increase in copy number at 1 h, 2 h, 3 h, and 16 h after treatment with mitomycin C. The statistical significance of the increase in RD2 copy number was determined by t-test, *** on the graph denotes p value below 0.001.

To further investigate the putative presence of multiple extrachromosomal copies of RD2 in GAS cells, we performed quantitative real time PCR using total DNA isolated from MGAS6180 strain. Performed analysis revealed that RD2 is present in 6-9 copies per chromosome (Figure 5B, see below). Also, the amplification of chromosomal junction (primers #1+#4) suggests that RD2 can be excised from the site of integration.

Taken together, these results indicate that a circular form of RD2 is present in strain MGAS6180.

### Response of strain MGAS6180 to mitomycin C and hydrogen peroxide treatment

We hypothesized that the putative circular form detected in overnight cultures (see above) is a transient form involved in DNA transfer. DNA damaging factors as ultraviolet light, hydrogen peroxide, or mitomycin C can induce mobilization of genetic elements such as prophages or pathogenic islands as part of a response to DNA damage or oxidative stress [23]. To test hypothesis that RD2 was induced/excised by DNA damage and oxidative stress, we examined induction of RD2 and five other integrative elements present in the genome of strain MGAS 6180 by mitomycin C and hydrogen peroxide treatment.

In the absence of mitomycin C, no significant increase in target gene copy number of any of tested element occurred, even after overnight incubation (Figure 5B), and the copy number of the element remained constant. However, RD2 copy number increased by 1 h, 2 h, 3 h, and 16 h-post mitomycin C treatment (Figure 5D). Of note, we also detected increases in the copy number of genes encoded by several other integrative elements present in the genome of strain MGAS6180. For example, all three tested prophages were induced. In the most dramatic case of prophage 6180.2 (encoding SpeK, a superantigen, and SlaA, a secreted phospholipase A_2 _virulence factor) we observed a increase in relative copy number over 700 times compared with the pre-induction level (Additional File 7, Figure S3). Consistent with phage induction, mitomycin C treatment resulted in a rapid decrease in optical density of the culture, presumably corresponding to cell lysis (Figure 5A).

Treatment with hydrogen peroxide did not increase RD2 copy number (Figure 5C), however we observed induction of phages such as 6180.1 and 6180.2 (Additional File 7 Figure S3).

### An RD2-like element is present in group C and G *Streptococcus *strains

Inasmuch as genome sequence information (Figure 1) and filter-mating data presented herein suggested that RD2 or an RD2-like element can spread between streptococcal species and multiple serotypes, we tested the hypothesis that the RD2 element has a phylogenetic distribution broader than GAS and GBS. To test the hypothesis, we screened 20 group C (GCS) and G (GGS) streptococci causing human infections by PCR for the presence of seven RD2 genes encoding putative extracellular secreted proteins. The primers and conditions we used were based on the sequence of RD2 found in GAS strain MGAS6180, and have been used previously to study the distribution of RD2 in GAS strains [1]. Because specific primers were used, this PCR analysis tests for the presence of genes with high homology to the RD2 element in GAS. The majority of the 20 GCS and GGS strains tested have homologs of RD2 element genes (Table 2A). DNA sequencing of all PCR products confirmed that the amplified gene fragments were homologues of RD2 element genes (data not shown). To test the hypothesis that the amplified genes were organized in an RD2-like genetic element, we used PCR primers described previously to tile across the entire RD2 element found in GAS strains [1]. The results (Table 2B) show that two GGS strains had an intact RD2 element, and one GCS strain had large segments of an intact RD2. The analysis also revealed a similar organization to RD2 in MGAS6180, as amplicons of the same size were generated (data not shown). Missing products of tiling PCR of GCS encompass homologs of M28_Spy1325 and M28_Spy1326 (fragments 9-10) which genes detected in single PCR reactions (Table 2A). The failure to amplify PCR products corresponding to the junction sites between the chromosome and RD2 suggests that the element is located in a different chromosomal location than in GAS. However, we cannot rule out the possibility that DNA sequence divergence at the primer pairing site was responsible for the lack of amplification of the target amplicon.

**Table 2:** Detection of RD2 element genes in Lancefield group C and G streptococci by PCR.

A. Detection of genes encoding putative extracellular proteins													
**Strain**	**M28_ Spy1306**	**M28_ Spy1307**		**M28_ Spy1308**		**M28_ Spy1325**		**M28_ Spy1326**		**M28_ Spy1332**		**M28_ Spy1336**	

GCS													

15169	**+**	**+**		-		**+**		**+**		-		**+**	

15170	**+**	**+**		-		-		**+**		-		-	

15172	**+**	**+**		**+**		**+**		**+**		-		**+**	

15173	**+**	**+**		-		**+**		**+**		-		**+**	

15178	**+**	**+**		**+**		**+**		**+**		**+**		**+**	

15181	**+**	**+**		-		**+**		**+**		-		**+**	

GGS													

15163	**+**	**+**		-		**+**		**+**		-		**+**	

15164	**+**	**+**		-		**+**		**+**		-		**+**	

15165	**+**	**+**		-		**+**		**+**		-		**+**	

15166	**+**	**+**		-		**+**		**+**		-		**+**	

15167	**+**	**+**		-		**+**		**+**		-		**+**	

15168	**+**	**+**		-		**+**		**+**		-		**+**	

15171	**+**	**+**		**+**		**+**		**+**		-		**+**	

15174	**+**	**+**		**+**		**+**		**+**		**+**		**+**	

15175	**+**	**+**		**+**		-		-		-		-	

15176	**+**	**+**		-		**+**		**+**		-		**+**	

15177	**+**	**+**		-		**+**		**+**		-		**+**	

15179	**+**	**+**		-		**+**		**+**		-		**+**	

15180	**+**	**+**		**+**		**+**		**+**		-		**+**	

15182	**+**	**+**		**+**		**+**		**+**		**+**		**+**	

**B**. PCR-tiling across the entire RD2 element. Example of the tiling across RD2 is presented in Figure 3. (+) PCR product present, (-) no product, * amplified fragment of different size than for strain MGAS6180													

		**PCR-tiling fragment no.**											

**Strain**	**group**	**1**	**2**	**3**	**4**	**5**	**6**	**7**	**8**	**9**	**10**	**11**	**12**

6180	A	**+**	**+**	**+**	**+**	**+**	**+**	**+**	**+**	**+**	**+**	**+**	**+**

15178	C	-	**+**	**+**	**+**	**+**	-	-	**+**	-	-	**+**	-

15174	G	+(*)	**+**	**+**	**+**	**+**	**+**	-	**+**	**+**	**+**	**+**	-

15182	G	-	**+**	**+**	**+**	**+**	**+**	**+**	**+**	**+**	**+**	**+**	-

## Discussion and Conclusions

Analysis of multiple genomes of GAS shows that about 10% of the genome can be attributed to genetic material acquired horizontal gene transfer [3]. Multiple mobile genetic elements as prophages, ICE elements and ancient pathogenicity islands are part of GAS metagenome [3,24]. Lack of detected natural transformation of GAS, despite proposed mechanism mediated via quorum sensing mechanism, [25] stresses the importance of transduction and conjugation processes in HGT.

Since late 1970s multiple authors were studying plasmid conjugal transfer between various streptococcal species [26-28]. Later, based on sequence analyses and experimental rationale, horizontal transfer of genes/regions between GAS and GGS was implied [29-31]. Finally, recent publications report conjugative transfer of ICE elements in human and animal isolates of GAS, GBS, GGS, GCS and *Streptococcus uberis *[32,33].

Our work demonstrates that genetic element RD2 from GAS strain MGAS6180 (serotype M28) can be horizontally transferred in the laboratory to other GAS strains by filter mating. The transfer frequency is comparable with inter-species transfer of ICE*St3 *[34]. However, we cannot exclude that the transfer frequency was influenced by the inactivation of M28_Spy1325-1326 genes. The genes encode putative extracellular proteins and can act as aggregation factors, in particular, M28_Spy1325 has homology to enterococcal conjugative plasmid pAM373 aggregation factor [35]. However, because we used filter mating technique that can at least partially circumvent the need of aggregation factor in the conjugation process, the lack of M28_Spy1325-1326 genes does not have to affect transfer frequency during filter mating.

Presented study provides experimental support for the idea that the presence of RD2 in GAS strains of very diverse phylogenetic backgrounds that have not shared a recent common ancestor have acquired this element by lateral gene flow occurring in nature. Our results also show that RD2-like regions are present in multiple Lancefield group C and group G strains, additional evidence for horizontal dissemination of RD2 in natural populations of streptococci. Of note, the detection of an RD2-like element in group B [16], C and G streptococci (this work) is consistent with early reports of the production of the R28 antigen in these organisms [5,36].

We believe that RD2 has spread and been maintained in genetically diverse organisms in part because proteins encoded by this genetic element confer a survival advantage to the recipient organism. RD2 encodes at least seven proteins that are secreted into the extracellular environment, including several likely to participate in host-pathogen interactions such as cell adhesion. It is plausible that at least two of these proteins confer a survival premium. The best characterized is protein R28 encoded by M28_Spy1336. The RD2 protein has been shown to promote adhesion of GAS to human epithelial cells grown *in vitro *and confer protective immunity in a mouse model of invasive disease, together providing evidence that the R28 protein is a virulence factor [5,6]. Another RD2 encoded gene involved in virulence is M28_Spy1325. The protein is a member of the antigen I/II family of adhesions made by oral streptococci. It is made *in vivo *during invasive GAS infection, and binds GP340, a heavily glycosylated protein present in human saliva [8]. Similar to the R28 protein, immunization with recombinant purified M28_Spy1325 protect mice from experimental invasive infection, and the protein is made during human invasive infections [1,8]. Although far less is known about the other secreted extracellular proteins made by RD2, serologic analysis indicates that M28_Spy1306, M28_Spy1326 and M28_Spy1332 also are made during human invasive infections [1].

Although our work did not define the exact molecular mechanism(s) mediating horizontal gene transfer of RD2, the structure of the element and its transfer by filter mating point toward conjugation as a key process. Parts of RD2 share substantial homology with ICE*St1 *[37] and ICE*St3 *[38] conjugative elements from *S. thermophilus*. ICE*St1 *and ICE*St3 *elements have homology in sequence and organization with conjugative transposon Tn*916 *from *Enterococcus faecalis *[39]. Interestingly, a large intergenic region between M28_Spy1321 and M28_SpyM28_Spy1322 ORFs contains multiple palindromic sequences and might function as origin of transfer (*oriT*) as the equivalent region of Tn*916 *has been shown [40] or has been suggested to function as such [18].

We note that the copy number of RD2 increased rapidly and substantially following treatment of strain MGAS6180 with mitomycin C, a DNA damaging agent known to induce the SOS response and stimulate horizontal transfer of ICEs [23,41-43].

Interestingly, Ubeda et al. have reported that other factors as antibiotic treatment can mediate SOS response in staphylococci and promote horizontal dissemination of pathogenicity island-encoded virulence factor genes [44]. The postulated mechanism of SOS-induced induction and transfer of ICE*St1/3 *elements involves autoproteolysis of cI type repressor Arp1 [23,45]. As the RD2 element encodes multiple cI type repressors [1] it is plausible that the mechanism of RD2 induction is mediated by SOS-induced proteolysis or autoproteolysis of one of the RD2 cI regulators. The induction of RD2 was not observed after treatment with hydrogen peroxide i.e. in the condition of oxidative stress that is known to induce phages [46-48]. That suggests rather LexA dependent mechanism induced by DNA damage.

In conclusion, RD2 is a medium host range mobile element that is shared between multiple unrelated serotypes of GAS and other pathogenic streptococcal species. As a consequence of several extracellular secreted proteins encoded by RD2, the element may confer a selective advantage on organisms that acquire this element by horizontal gene transfer.

## Authors' contributions

IS conceived, designed, coordinated the study and wrote the manuscript; performed the bioinformatics analysis of RD2 region, filter mating experiments and analysis of gene copy number. NMG participated in the design of the study, analysis of the results and wrote the manuscript; performed the bioinformatics analysis of RD2 region; screened GCS and GGS strains for the presence of RD2 element and constructed the RD2 mutant.

NG detected multiple RD2 copies. LM participated in data analysis, and screened GCS/GGS strains for the presence of RD2 element. JMM analyzed the data and wrote the manuscript. All authors read and approved the final manuscript.

## Supplementary Material

Additional file 1 Table S1: Streptococcal strains used in the study Click here for file

Additional file 2 Table S2: Primers used for the mutant construction Click here for file

Additional file 3 Supplemental Methods Click here for file

Additional file 4 Figure S1: Conformation of proper mutant construction Click here for file

Additional file 5 Figure S2: Determination of MIC values for mitomycin C and hydrogen peroxide Click here for file

Additional file 6 Table S3: Homologs of RD2 genes found in GBS Click here for file

Additional file 7 Figure S3: Induction of prophages and ICE elements in MGAS6180 after treatment with mitomycin C and hydrogen peroxide. Click here for file
