# Supplementary material for: Lateral gene transfer of streptococcal ICE element RD2 (region of difference 2) encoding secreted proteins
Source: BMC Microbiol. 2011 Apr 1;11:65. doi: 10.1186/1471-2180-11-65 (PMC3083328; doi:10.1186/1471-2180-11-65)
Supplement: Additional file 1 — Table S1: Streptococcal strains used in the study [file 1471-2180-11-65-S1.DOC]

**Additional File 1, Table S1 Streptococcal strains used in the study.**

| **Strain** | **Genotype/serotype** | **Source/reference** |
| --- | --- | --- |
| Group A *Streptococcus* | | |
| MGAS6180 | M28 | [1] |
| MGAS6180Δ1325-1326spc^R^ | M28 | This work |
| MGAS9455 | M28 | Musser lab collection |
| MGAS9538 | M28 | Musser lab collection |
| MGAS10208 | M28 | Musser lab collection |
| MGAS11932 | M2 (previously typed as M124) | Musser lab collection |
| MGAS10270 | M2 | [2] |
| MGAS10750 | M4 | [2] |
| MGAS10218 | M2 | Musser lab collection |
| MGAS10422 | M2 | Musser lab collection |
| MGAS9482 | M77 | Musser lab collection |
| MGAS5005 | M1 | [3] |
| MGAS2221ΔcovRS | M1 | P. Sumby, unpublished |
| Group C *Streptococcus* | | |
| MGCS15169 | --- | Musser lab collection |
| MGCS15170 | --- | Musser lab collection |
| MGCS15172 | --- | Musser lab collection |
| MGCS15173 | --- | Musser lab collection |
| MGCS15178 | --- | Musser lab collection |
| MGCS15181 | --- | Musser lab collection |
| Group G *Streptococcus* | | |
| MGGS15163 | --- | Musser lab collection |
| MGGS15164 | --- | Musser lab collection |
| MGGS15165 | --- | Musser lab collection |
| MGGS15166 | --- | Musser lab collection |
| MGGS15167 | --- | Musser lab collection |
| MGGS15168 | --- | Musser lab collection |
| MGGS15171 | --- | Musser lab collection |
| MGGS15174 | --- | Musser lab collection |
| MGGS15175 | --- | Musser lab collection |
| MGGS15176 | --- | Musser lab collection |
| MGGS15177 | --- | Musser lab collection |
| MGGS15179 | --- | Musser lab collection |
| MGGS15180 | --- | Musser lab collection |
| MGGS15182 | --- | Musser lab collection |

1. Green NM, Zhang S, Porcella SF, Nagiec MJ, Barbian KD, Beres SB, LeFebvre RB, Musser JM: **Genome sequence of a serotype M28 strain of group A *Streptococcus*: potential new insights into puerperal sepsis and bacterial disease specificity**. *J Infect Dis* 2005, **192**(5):760-770.

2. Beres SB, Richter EW, Nagiec MJ, Sumby P, Porcella SF, DeLeo FR, Musser JM: **Molecular genetic anatomy of inter- and intraserotype variation in the human bacterial pathogen group A *Streptococcus***. *Proc Natl Acad Sci U S A* 2006, **103**(18):7059-7064.

3. Sumby P, Porcella SF, Madrigal AG, Barbian KD, Virtaneva K, Ricklefs SM, Sturdevant DE, Graham MR, Vuopio-Varkila J, Hoe NP *et al*: **Evolutionary origin and emergence of a highly successful clone of serotype M1 group A *Streptococcus* involved multiple horizontal gene transfer events**. *J Infect Dis* 2005, **192**(5):771-782.
