# Supplementary material for: Lateral gene transfer of streptococcal ICE element RD2 (region of difference 2) encoding secreted proteins
Source: BMC Microbiol. 2011 Apr 1;11:65. doi: 10.1186/1471-2180-11-65 (PMC3083328; doi:10.1186/1471-2180-11-65)
Supplement: Additional file 2 — Table S2: Primers used for the mutant construction [file 1471-2180-11-65-S2.DOC]

**Additional File 3, Table S2 Primers used for the mutant construction**

| **Primer name** | **Primer sequence** |
| --- | --- |
| 5’_1325for | CCATAACCCTTAGTTTCCATTTGTTTC |
| 5’ 1325rev | GGCTAACCTAACAGCACAGTATCAAGCTATC |
| 3’_1325for | CCAACAACAAGTCCACGTGATTCG |
| 3’_1325rev | CTACCCGGGCTCTCACTCTTAGGTTATGTAGGGC |
| 5’_1326for | GCTGCGACTGCGATTCCTGTTAATAC |
| 5’_1326rev | GTATGCTCGAGATAGAGGCAACAAC |
| 3’_1326for | GCATCTACTTGGGCTTGACCAGTTTC |
| 3’_1326rev | CATCCCGGGGCTTAGGAAGCAAGAGAAAAG |
| Spcrev | TTATAATTTTTTAATCTGTTATTTAAATAGTTTATAG |
| Spcstart | ATGTTTGGATCAGGAGTTGAGAGTGGAC |
