# Supplementary material for: Lateral gene transfer of streptococcal ICE element RD2 (region of difference 2) encoding secreted proteins
Source: BMC Microbiol. 2011 Apr 1;11:65. doi: 10.1186/1471-2180-11-65 (PMC3083328; doi:10.1186/1471-2180-11-65)
Supplement: Additional file 3 — Supplemental Methods [file 1471-2180-11-65-S3.DOC]

**Supplemental materials and methods**

**Generation of plasmid constructs for the replacement of 1325-1326 genes with spc^R^ cassette.** The oligonucleotide primers used for plasmid construction are listed in Supplemental Table 1. DNA fragments encoding the upstream and downstream region of *spy1325 and spy1326,* were amplified by PCR and cloned separately into PCRII-TOPO (Invitrogen) to create p1325-3’, p1325-5’, p1326-3’ and p1326-5’plasmids. Plasmids containing the downstream regions for each coding sequence (p1325-3’, p1326-3’) were linearized by *SmaI* digestion and ligated to *SmaI*-digested spectinomycin (spc) resistance cassette excised from pSL60-2 (16) creating p1325-3’-spc, p1326-3’-spc. *EcoRI* digestion followed by gel isolation was used to isolate these fragments containing spc cassette and GAS insert from each plasmid. Klenow treatment was used to blunt the *EcoRI* ends of the fragments before ligation to *EcoRV*-digested plasmids containing the upstream flanking regions (p1325-5’, p1326-5’). Orientation of cloned regions at each step was determined by PCR. p1325-3’-spc and p1326-5’ were combined to generate p1325-1326. Briefly, plasmid p1325-3’-spc was digested with *EcoRI* to isolate the fragment containing the spc cassette and GAS insert. Klenow treatment was used to blunt the *EcoRI* ends of the fragment before ligation to *EcoRV*-digested p1326-5’. Orientation of cloned regions at each step was determined by PCR. Plasmid DNA from the final recombinant construct, p1325-1326, was used for PCR amplification of a fragment containing flanking regions and spectinomycin cassette. Purified PCR product (QIAquick PCR purification kit, Qiagen) was used to transform electrocompetent GAS cells.

**Western immunoblot analysis.** Wild-type strain MGAS6180 and the isogenic mutant derivative strain were grown to stationary phase, and cells were pelleted by centrifugation. Proteins from the cell culture supernatant were precipitated by adding 30% ice-cold trichloroacetic acid (TCA) (Sigma-Aldrich) and incubated on ice for 1 h. The precipitated proteins were collected by centrifugation at 18,000 x g for 15 min at 4°C, washed twice with acetone, dried under vacuum, and suspended in phosphate buffered saline. The precipitated proteins were analyzed with 7.5% Tris-HCl gels (BioRad), transferred to nitrocellulose membranes, and incubated with a 1:3000 dilution of rabbit polyclonal anti-Spy1325 antibody raised against purified recombinant Spy1325 . The secondary antibody was goat anti-rabbit HRP-conjugate (Pierce) used at a dilution 1:10,000.

**Determination of minimal inhibitory concentration of mitomycin C and hydrogen peroxide (MIC) for MGAS6180**. 200 μl of THY medium with 0,8 μg/ml of mitomycin C was twofold diluted in columns of sterile 96 well plate to reach the concentration 0,4ng/ml. Each of the wells was inoculated 1:50 with overnight culture of MGAS6180.

200 μl of THY medium with 2mM hydrogen peroxide was twofold diluted in columns of sterile 96 well plate to reach 1nM concentration. Optical density was checked every hour for 8 hours and after 24h of growth. MIC was the last concentration that inhibited growth of bacteria. Assay for each concentration of tested substance was replicated at least 3 times
