# Supplementary material for: Lateral gene transfer of streptococcal ICE element RD2 (region of difference 2) encoding secreted proteins
Source: BMC Microbiol. 2011 Apr 1;11:65. doi: 10.1186/1471-2180-11-65 (PMC3083328; doi:10.1186/1471-2180-11-65)

**Additional File 4, Figure S1.** Confirmation of the proper construction of MGAS6180Δ1325-1326 mutant. **A**. PCR amplification of modified chromosomal region in mutant. Amplified fragments:1. flank of M28_1325; 2. spc cassette; 3. flank of M28_1326; 4. fragment upstream integration site + flank of M28_1325; 5. flank of M28_1325 + spc cassette; 6. flank of M28_1326 + spc cassette; 7. fragment downstream integration site + flank of M28_1326; 8. Fragment encompassing chromosomal region from the upstream of integration site to spc cassette; 9. Fragment encompassing chromosomal region from the downstream of integration site to spc cassette; 10. Amplification across the whole integrated casette

**B**. Western blot detection of extracellular production of M28_Spy1325 by WT and the mutant.


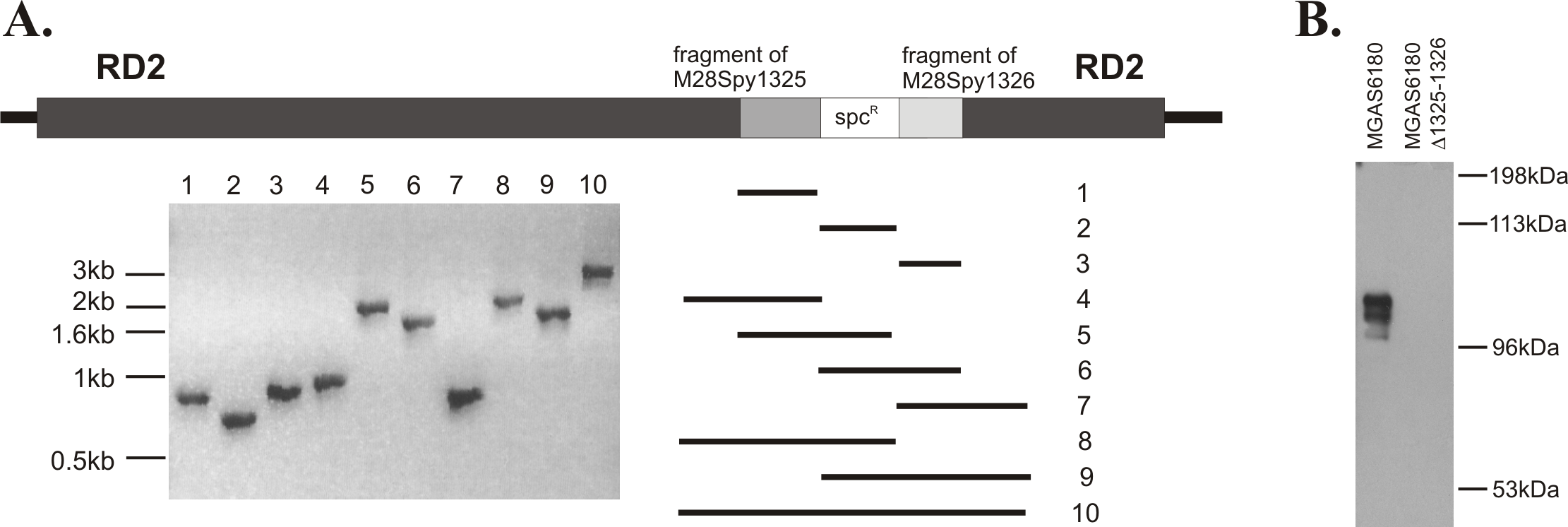

Supplement: Additional file 4 — Figure S1: Conformation of proper mutant construction [file 1471-2180-11-65-S4.DOC]
