# Supplementary figures and images for: Lateral gene transfer of streptococcal ICE element RD2 (region of difference 2) encoding secreted proteins
Source: BMC Microbiol. 2011 Apr 1;11:65. doi: 10.1186/1471-2180-11-65 (PMC3083328; doi:10.1186/1471-2180-11-65)

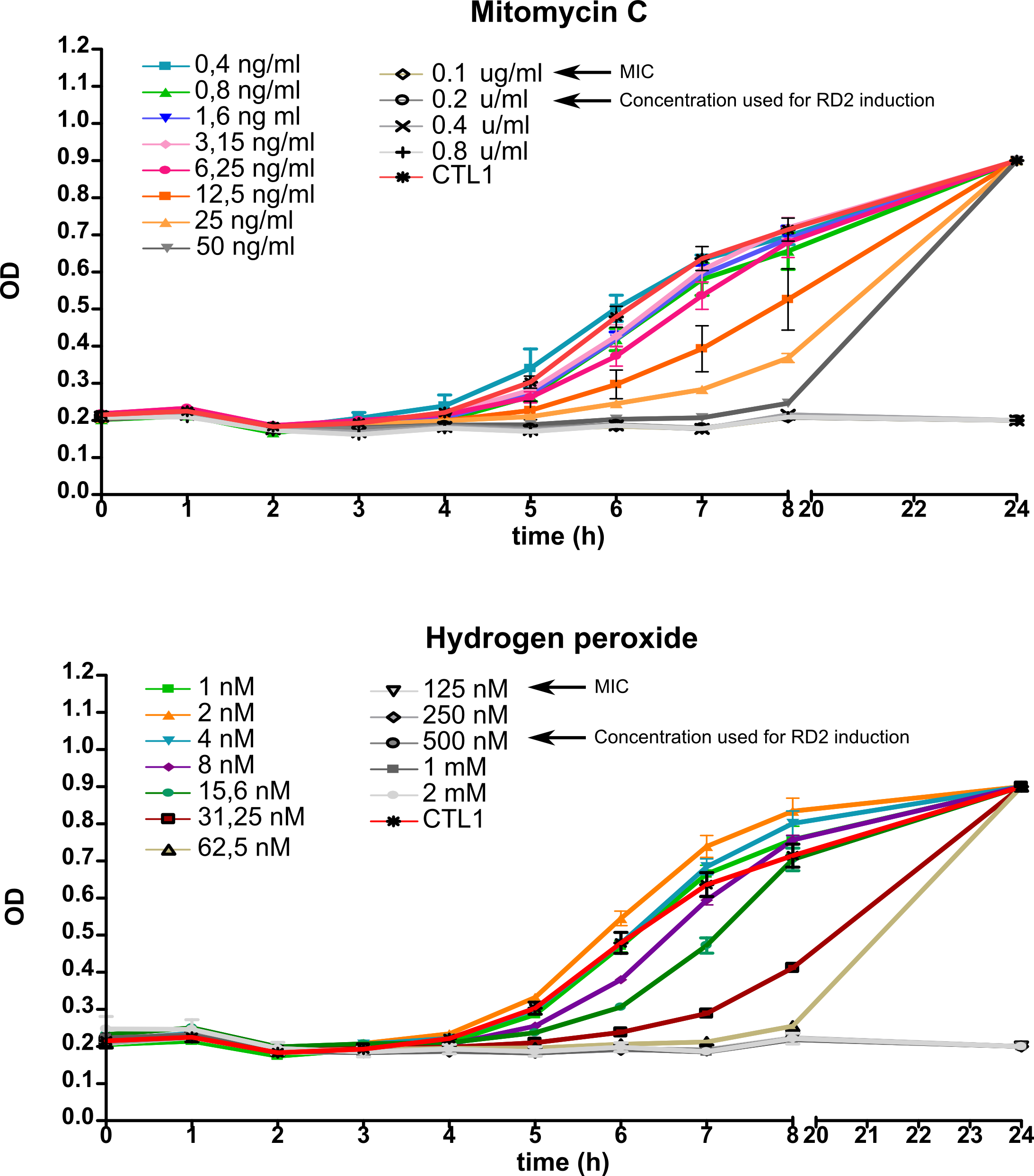

Supplement: Additional file 5 — Figure S2: Determination of MIC values for mitomycin C and hydrogen peroxide [file 1471-2180-11-65-S5.PNG]

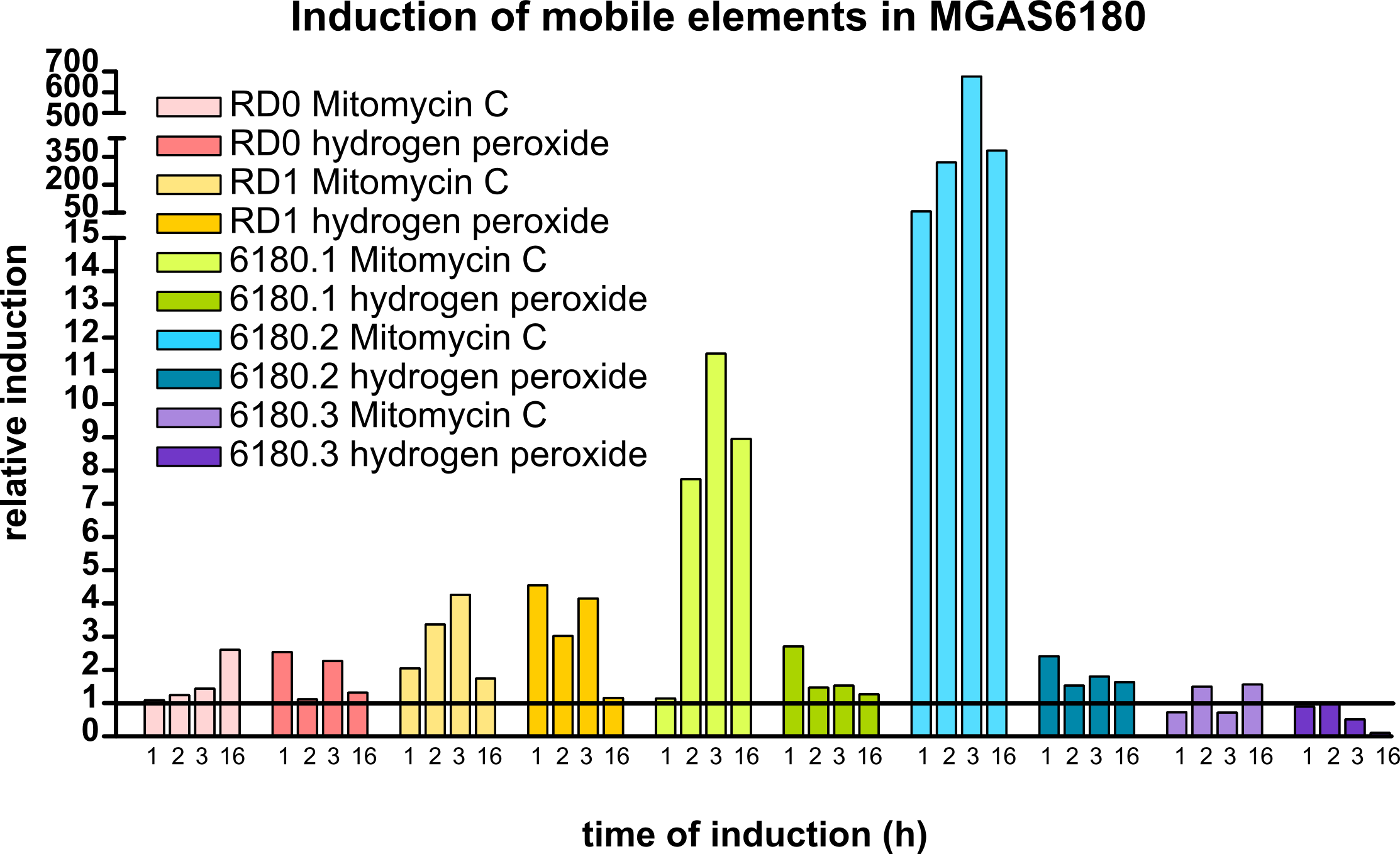

Supplement: Additional file 7 — Figure S3: Induction of prophages and ICE elements in MGAS6180 after treatment with mitomycin C and hydrogen peroxide. [file 1471-2180-11-65-S7.PNG]
